# Effectiveness of home-based telerehabilitation programs on functional capacity and cardiac function in elderly heart failure patients: A prospective longitudinal study

**DOI:** 10.1097/MD.0000000000029799

**Published:** 2022-07-15

**Authors:** Wei-Jung Tsai, Yan-Kai Wen, Yuan-Yang Cheng, Jin-Long Huang, Yan-Wen Chen

**Affiliations:** a Department of Physical Medicine and Rehabilitation, Taichung Veterans General Hospital, Taichung, Taiwan; b School of Medicine, National Yang-Ming University, Taipei, Taiwan; c Cardiovascular Center, Taichung Veterans General Hospital, Taichung, Taiwan; d Institute of Clinical Medicine, and Cardiovascular Research Institute, Department of Medicine, School of Medicine, National Yang-Ming University, Taipei, Taiwan.

**Keywords:** cardiac function, elderly, functional capacity, heart failure, home-based cardiac telerehabilitation, post-acute care

## Abstract

Decreased functional capacity and reduced cardiac function were the main symptoms in patients with heart failure (HF) and the incidence increases with advanced age. The guidelines recommend that exercise training should be considered for medically stable HF outpatients. Studies have confirmed that exercise can improve functional capacity, prognosis, and reduced hospitalization rates; however, very few studies have investigated the elderly. It is not clear whether exercise could be feasible in elderly HF. The aim of this study was to evaluate the effect of the 6-month heart failure post-acute care program focused on home-based cardiac telerehabilitation (HCTR) on functional capacity, cardiac function, and readmission rates in HF patients.

A prospective longitudinal study was conducted. Study duration was from January 2018 to December 2019. HF patients with a left ventricular ejection fraction <40% and age ≧65 years were included and divided into intervention (n = 40) and control group (n = 41). We arranged a 6-month heart failure post-acute care program that included outpatient cardiac rehabilitation and home exercise for the intervention group. The response to home exercise was followed by telemonitor. The exercise parameters were recorded on the HF health management mobile application system platform by each patient and daily transmission to hospital’s cloud database as HCTR, usual care program for the control group. Information such as general data, laboratory data, six-minute walk test, cardiac function, and admission record was collected from all patients.

Eighty one patients between the ages of 65 and 92 completed the study. The mean age was 73.3 ± 5.0 and 75.6 ± 6.0 years in control and intervention group, respectively. The intervention group showed a statistically significant improvement in functional capacity, percentage change in the of six-minute walk distance (51.2% vs 17.7%, *P* < .05, 95% confidence interval −45.9 to −6.3). Left ventricular ejection fraction increased by 7.7%, which corresponds to 25.6% in relative terms (*P* < .05, 95% confidence interval −7.8 to −0.5). The readmission rate was 4.6% in the intervention group.

Six months of post-acute HF focused on HCTR programs was safe, improved functional capacity, cardiac function, and decreased readmission rate in elderly patients with HF patients.

## 1. Introduction

Heart failure (HF) is a clinical syndrome of ventricular dysfunction associated with exercise intolerance, breathiness, and fatigue.^[1]^ Its prevalence increases with age, rising to >10% among individuals age ≧70 years and 50% among those aged ≧80 years.^[2,3]^ Reduced physical function is one of the most common complaints among those diagnosed with HF.^[4]^ Bedsides, impaired exercise capacity, reduce daily activity, which in the elderly is exacerbated by significant amount of comorbidities. It is highly associated with hospitalization, falls, and the level of dependency on family and care givers.^[5]^ Several studies have shown that evidence-based HF treatment that included exercise intervention can improve exercise tolerance, prognosis, and reduced hospitalization rates in these patients, however, very few studies have investigated elderly patients.^[6–9]^ Exercise intervention is an important adjunct nonpharmacologic treatment modality for HF patients and is recommended by the American College of Cardiology Foundation/American Heart Association, the European Society of Cardiology.^[6,10,11]^ In addition, several studies have demonstrated the value of remote telemonitoring in the management of HF patients. However, the effectiveness of home-based cardiac telerehabilitation (HCTR) in elderly patients has not been widely studied and its training effects remain unclear.

Therefore, the objective of this study was to evaluate the effects of the HCTR program in patients with post-acute decompensate HF. We hypothesized that our HCTR programs are superior to usual care to improve functional capacity and cardiac function. The primary outcome was functional capacity assessed by the six-minute walk distance (6MWD); secondary outcome was cardiac function assessed by left ventricular ejection fraction (LVEF) and readmission rates.

## 2. Methods

### 2.1. Study design and procedure

This study was a prospective longitudinal study, a total of 139 patients admitted due to acute decompensation HF were invited to enroll in the Heart failure post-acute care (HF-PAC) after discharge from January 2018 to December 2019.

HF-PAC is a multidisciplinary integrated case management program for outpatients. The specialists included cardiologist, pharmacist, physical therapist, psychologist, dietitian, and case managers specialized in HF. Participants divided into an intervention group or a control group. The intervention group attended supervised outpatient cardiac rehabilitation exercise training and the control group received usual medical care. The clinical data of the study were collected by case managers and physical therapists. The study was then conducted after obtaining permission from the hospital institutional review board.

### 2.2. Participants and setting

The HF-PAC case manager recruited patients due to acute decompensation HF at Taichung Veterans General Hospital were recruited by the HF-PAC case manager to enroll in the study. Age ≧65 years old diagnosed with acute decompensated HF and discharged from the hospital within 2 weeks were eligible to enroll. Other inclusion criteria were (1) New York Heart Association functional class II to IV; (2) LVEF ≤ 40%; (3) American College of Cardiology/American Heart Association classification of heart failure class C–D; (4) with stable medical therapy. Exclusion criteria included long-term mechanical ventilator dependent, hemodialysis, severe cognitive impairment, bedridden >3 months, underwent coronary artery bypass graft or valve surgery within 1 month, and patient refused due to difficulty performing evaluation.

**Figure 1.**
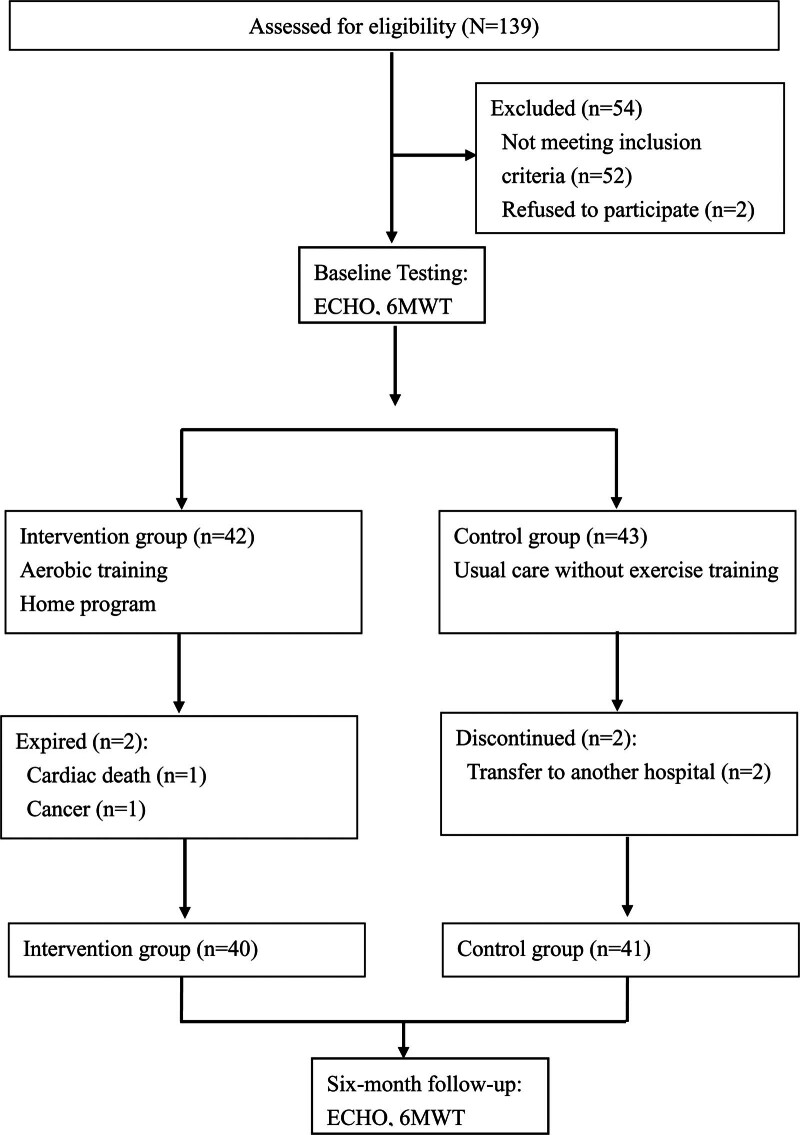
Participant’s flow chart and follow-up. 6MWT = six-minute walk test, ECHO = echocardiography.

Initially, 139 patients were considered potentially eligible to participate in the study. Fifty two were excluded. 2 patients declined to participate due to lack of time. Of the remaining 85 participants, 2 dropped out of the intervention group due to expired and 2 dropped out in the control group due to personal reasons. Therefore, a total of 81 patients completed the study: 41 patients in the control group, 40 patients in the intervention group (Fig. 1). A case manager collected the measurements and baseline characteristics (e.g., demographics, laboratory data, echocardiogram, and six-minute walk test [6MWT]). The Gpower program was used to calculate and estimate the sample size for the study (actual power = 75%).^[12]^

### 2.3. Measurements

Basic data included age, gender, body weight, height, body mass index, medications, and laboratory data within the study. The 6MWT and LVEF were measured at baseline and 6 months later to evaluate functional capacity and cardiac function in these patients with HF. Our primary outcome was the functional capacity evaluated by 6MWT. After informing the patients about the aim of the test, the measurement followed the guidelines of the American Thoracic Society.^[13]^ The patients were instructed to walk along a 30-m corridor. They were told to walk continuously, however, at their limits they could slow down or stop if necessary. The test was performed by the same physical therapist. The outcome measure was the total walking distance covered in 6 minutes; distance covered was recorded in meters. According to Hamilton et al, which examine the 6MWT is a valid and reliable method to assess functional ability in a cardiac rehabilitation population, and have been found to be moderately with peak VO2 in exercise testing in patients with HF, also a fairly accurate predictor of increased mortality and morbidity of heart disease.^[13,14]^

Our secondary outcome was cardiac function evaluated by LVEF and readmission rate. To evaluate heart function, an echocardiogram was used for calculated LVEF. Transthoracic echocardiography was performed by trained cardiologists. LVEF was calculated using the biplane Simpson method on apical 4- and 2-chamber views as recommended by the American Society of Echocardiology and the Taiwan Society of Cardiology Guidelines for HF. The readmission rate was defined as rate of all-cause associated rehospitalization within 30 day and 6 months during follow-up. Safety is defined as any exercise-induced adverse event during telemonitoring.

### 2.4. Intervention

Participants were requested not to participate in any other exercise programs during the 6-month period. After the acute phase of the inpatient, if the patient is a reversible disease and still needs care, he is transferred to the community or home to receive continuous care without continuing to be hospitalized. During the treatment period, patients will receive intense rehabilitation and integrated care according to the individualized treatment program devised by the medical team. The intervention group attended the cardiac rehabilitation exercise at the Department of Rehabilitation for supervised exercise training by a physical therapist and performed home exercise as a prescript. A single session consisted of 40 to 60 minutes of exercise including warm-up and cool-down in total. The exercise intensities setting of the resting heart rate plus 30 beats per minute. Aerobic exercise includes cycle ergometers, stepper, elliptical cross trainers, walking training. Heart rate, blood pressure, 3-lead electrocardiogram recordings, arterial O_2_ saturation, and rated perceived exertion (6–20 scale) were monitored. The increase in intensity of training was based on individual tolerability and improvement (e.g., rated perceived exertion below somewhat hard, 13/20). The control group received usual care including basic nursing, medication, nutrition, and exercise education before discharge from the hospital after the acute inpatient phase. The control group could join outpatient cardiac rehabilitation after the last follow-up meeting.

**Figure 2.**
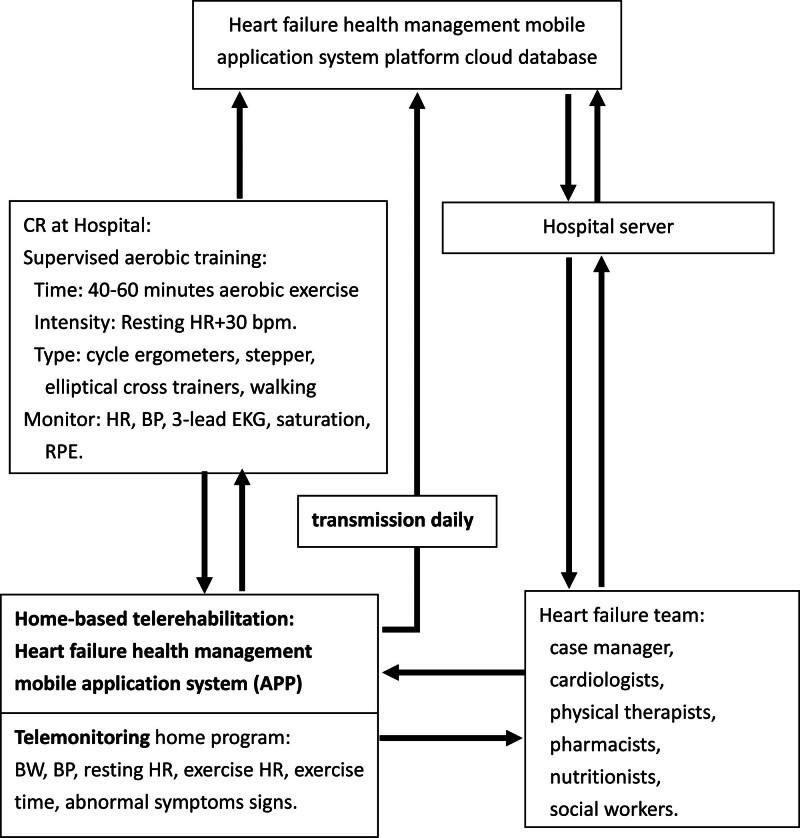
Schema of detailed intervention and exercise parameters in the intervention group. BP = blood pressure, BW = body weight, CR = cardiac rehabilitation, EKG = electrocardiogram, HR = heart rate, RPE = rated perceived exertion.

The response to home exercise was followed by telemonitor. Monitoring parameters include body weight, blood pressure, resting heart rate, exercise heart rate, exercise time, and abnormal symptom signs were recorded on the HF health management mobile application system platform by each patient and regular daily routine transmission to the hospital cloud database as HCTR (Fig. 2). The case manager and the physical therapists reviewed the data and found that the monitoring parameters were abnormal, immediately contact the patient, and return to the hospital for examination if necessary. The medications were not changed in any patient during the telerehabilitation course.

### 2.5. Ethical considerations

Institutional review board approval (Reference number: TCVGH-CE19077A, TCVGH-CF14356A-4) was obtained from the local medical center in Taiwan. All participants gave their informed written consent when invited to join the study.

### 2.6. Statistical analysis

Continuous variables were expressed as mean and standard deviation, categorical variables were expressed as number and percentage. Comparison between pre-training and post-training within the same group was made using the paired sample *t* test. The χ^2^ test was used for the comparison of categorical variables. The comparison between groups was made using the Student *t* test. *P* < .05 was accepted as statistically significant. SPSS 18.0 software (SPSS, Chicago, IL) was used for statistical analysis.

## 3. Results

### 3.1. Baseline characteristics of participants

Eighty-one patients between 65 and 92 years of age completed the study. The mean age was 73.3 ± 5.0 and 75.6 ± 6.0 years in control and intervention group, respectively. Men comprised more than half (67.5%–70.7%) of the sample. There were no demographic differences (age, body weight, disease etiology, left ventricular eject fraction, other medical history) between the groups, except history of hypertension. Sixty percent of the patients had hypertension in the intervention group and 31.7% hypertension in the control group. Diabetes mellitus and dyslipidemia were comparable in both groups. Table 1 presents the demographic and baseline physical characteristics including body weight, body mass index, and 6MWD. The number of dropouts was 2 in each group. The treatment compliance rate was 95.2% in the intervention group. The effect size of the the percentage change of 6MWD was 0.59. And the effect size of the improvement in the LVEF was also 0.59.

**Table 1:** Baseline characteristics of the participants.

Characteristics	Control (n = 41)	Intervention (n = 40)	*P*
Age (yr)	73.3 ± 5.0	75.6 ± 6.0	.07
Male	29 (70.7)	27 (67.5)	.81
Height (cm)	160.0 ± 7.3	160.9 ± 9.6	.12
BW (kg)	58.6 ± 11.9	63.3 ± 12.4	.19
BMI (kg/m^2^)	22.8 ± 3.4	24.1 ± 3.5	.09
LVEF (%)	33.5 ± 11.2	31.1 ± 10.5	.17
ICMP	5 (12.2)	9 (22.5)	.25
HTN	13 (31.7)	24 (60)	.01
DM	10 (24.4)	15 (37.5)	.24
Dyslipidemia	8 (19.5)	16 (40)	.06
6MWD (m)	250.8 ± 92.6	206.1 ± 104.9	.07

Data are presented as mean ± standard deviation or number (%).

6MWD = six-minute walk distance, BMI = body mass index, BW = body weight, DM = diabetes mellitus, HTN = hypertension, ICMP = ischemic cardiomyopathy, LVEF = left ventricular ejection fraction.

### 3.2. Primary outcome: Functional capacity

In the intervention group, the patients showed a significant improvement in functional capacity compared with control group after 6 months. There was a significant (*P* = .03) improvement in the percentage change in 6MWD in the intervention versus the control group at 6 months (51.2% vs 17.7%, *P* < .05, 95% confidence interval [CI] −45.9 to −6.3). 6MWD increased significantly after 6 months in both groups. The control group improved significantly from 250.8 ± 92.6 to 286.5 ± 99.3 m after 6 months, and the 95% CI was −54.63 to −5.82. It also improved significantly in the intervention group from 206.1 ± 104.9 to 272.4 ± 110.5 m, and the 95% CI was −86.23 to −52.11 (Table 2). The intervention group generates 51.2% versus 17.7% (*P* < .05, 95% CI −45.9 to −6.3), an average improvement of 63 m, and the control group an average improvement of 35 m. The comparison of 6MWD and changes between groups are presented in Table 2.

**Table 2:** Functional capacity and cardiac function at baseline and 6 mo in both groups.

Parameters	Control	Control, 6 mo	*P* value between CON groups	Change (%)	Intervention	Intervention, 6 mo	*P* value between INT groups	Change (%)	*P* value INT vs CON, 6 mo
Functional capacity									
6MWD (m)	250.8 ± 92.6	286.5 ± 99.3*	<.01	17.7	206.1 ± 104.9	272.4 ± 110.5*	.03	51.2**	.03**
Cardiac function									
LVEF (%)	31.1 ± 10.5	34.8 ± 13.3*	<.05	3.8	33.5 ± 11.2	38.8 ± 10.1*	.04	7.7**	.04**

Data are presented as mean ± standard deviation or as otherwise.

6MWD = six-minute walk distance, LVEF = left ventricular ejection fraction.

* *P* < .05 within group.

** *P* < .05 between groups.

### 3.3. Secondary outcome: Cardiac function, readmission rate

There was a significant improvement in the cardiac function in intervention group compared with control group after 6 months. The improvement in the percentage change in LVEF in the intervention versus control group at 6 months was 7.7% versus 3.8% (*P* < .05, 95% CI −7.8 to −0.5). LVEF in the control group improved from 31.1% ± 10.5% to 34.8% ± 13.3%, the 95% CI was −6.80 to −0.73; it also improved significantly in the intervention group from 33.5% ± 11.2% to 38.8% ± 10.1%. The comparison of LVEF between groups is presented as Table 2. The 30-day readmission rate for patients reduced from 13.6% to 4.1% after HCTR programs, and 6-month readmissions rate in the intervention group was 4.6%. There was no adverse event among the intervention group during the 6-month telemonitor.

## 4. Discussion

A major nonpharmacological treatment strategy in HF patients is cardiac rehabilitation exercise training. Our 6-month HF-PAC intervention was found to progressively increase functional capacity, cardiac function, and decrease readmission rates in elderly patients with HF. After 6 months of intervention, the functional capacity presented by changed in 6MWD improved 51%, the cardiac function presented by the LVEF increased 8%, 30-day and 6-month readmission rates at 30 days and 6 months were 4.1% and 4.6 %, while there was no significant change in the usual care group. Our results suggest that a combined HCTR is feasible and safe for elderly patients with HF.

Our primary outcome shows that the intervention group significantly increased 6MWD than the control group. Even both groups had improved 6MWD after 6 months. We found that the improvement in the percentage of change in the 6MWD was greater in intervention group. The meta-analysis offered strong evidence that 6MWD responded to the change in clinical status after cardiac rehabilitation, with an estimated mean distance difference in distance of 60.43 m.^[15]^ In our study, the results show that the intervention group generates an average improvement of 63 m, and the control group an average improvement of 35 m in elderly patients with HF. Therefore, in accordance with our results on functional capacity and exercise tolerance, it suggests a higher adaptation after the HF-PAC combine the HCTR programs.

To our knowledge, studies have been controversial about exercise training on cardiac function. Previous studies have found that exercise training does not offer benefits for heart function, including cardiac output, stroke volume, and LVEF.^[16–18]^ A published study showed that a 4-month period may be insufficient time to induce changes in LVEF.^[17]^ In our study, LVEF significantly improved 7% after 6 months of exercise training, which was similar to the study by Besnier et al^[19^ and Wisløff et al (9%).^[20]^ An improved cardiac function might suggest a decrease in patient severity.

Endothelial dysfunction contributes to enhanced peripheral vasoconstriction and impaired functional capacity in HF patient.^[21]^ Regular exercise improves muscle function, functional capacity and promotes the body’s ability to utilize oxygen.^[22]^ It also improves the capacity of blood vessels to dilate in response to exercise, left ventricular diastolic function, and neurohormone activation.^[23]^ During the immunologic and inflammatory responses that take place in the development and progression of HF, cytokines and oxygen-free radicals induce the expression of endothelial adhesion molecules.^[24]^ Shear stress is the most important physiological stimulus for nitric oxide generation. As a consequence of impaired left ventricular function and reduced blood flow in conductance and peripheral arteries in severe HF, less shear stress is exerted on the luminal surface of the endothelium. This results in lower endothelium-derived nitric oxide production and reduced endothelium-dependent dilation.^[25]^ Biological active N-terminal pro-brain natriuretic peptide is released from cardiomyocytes in response to myocardial wall tension.^[26]^ Exercise training has anti-inflammatory and antioxidant effects. It decreases the levels of circulating catecholamines and serum natriuretic peptides. It also increases shear stress and nitric oxide bioavailability. All of these lead to regular exercise training that could reduce peripheral vasoconstriction, improve endothelial function, and improve endothelial repair.^[27]^ This might explain the mechanism of improved functional capacity in our patients with HF.

Lower admission rates could reduce health-related costs. HF is a leading cause of hospital admission in the older adult, where it is associated with an increased hospital stay and risk of mortality. A significantly decreased readmission rates at 30 days and 6 months (4.1% and 4.6%) in our HF-PAC combine HCTR programs, compared to another hospital in Taiwan (5%–9.7%).^[28,29]^ HF is the most common cause of hospitalization in the United States for people older than 65 years of age, the 30-day rehospitalization rate, accounting for up to 26.9% of the total readmission rates.^[30]^ Targeted interventions, such as patient education and follow-up appointments, can help reduce 30-day readmission rates to 28% from 14% for patients with HF.^[31]^ In our study, the integrated HF-PAC and HCTR program could lower readmission rates compared to previous studies.

HF management and self-care behavior are complicated by aging, comorbid conditions, cognitive impairment, frailty, and limited social support. Due to complicated heart management and self-care, we used an easy mobile application to manage patient condition. Palmer et al enrolled 21 studies (n = 3082), but most of studies patients were under 65 years old which differing from our patients.^[32]^ Miles et al used a traditional exercise diary and the physical therapist reviewed the diary daily during the first 3 months. During 3 to 6 months, they exercised at home but without face-to-face contact with the physical therapist, the physical therapist reviewed the diary by telephone weekly.^[8]^ We use mobile application instead traditional diary so all the exercise parameters, vital signs were recorded in the system platform and regular daily transmission to hospital’s cloud database within 6 months. So, we could find out the patients with insufficient exercise very early and kept encourage them to include their family. The difference between HF-ACTION and our study was that their median age ranged from 59.3 to 59.2 years, our mean age were 73.3 to 75.6 years old, focus on elderly HF patients.

Research improved the idea that clinicians need to focus more on engaging patients with HF in rehabilitation to improving exercise adherence and compliance. This study could provide clinicians with confidence that the study is safe and effective in what is known to be a difficult population. Adherence to exercise is a particular challenge for patients with HF, who are often given multiple regimens, including fluid restriction and heavy pharmaceutical interventions.^[33]^

These elderly HF patients combined frailty in most of them, however, in the absence of a universal definition, measuring frailty has been challenging. However, we will add an evaluation of frailty in chronic elderly HF patients in the future study to better understand these patients.

The clinical significance of this study indicated that 6 months of post-acute HF with focusing a HCTR programs improved functional capacity (assessed by 6MWD), cardiac function (assessed by LVEF), and readmission rate in elderly HF with reduced ejection fraction. To elderly patients with HF, this study provided some evidence of safety in regular aerobic exercise and the effectiveness of telemonitoring for populations with reduced ejection fraction.

Based on our case number, the power of the the percentage change of 6MWD was 75%, and the power of the the change of LVEF was 73% calculated by G*power.^[12]^ It is not powerful enough to predict the results of all elderly patients with HF. This is a limitation of the study.

## 5. Limitations

A limited number of subjects were considered, and we only considered patients suffering from HF with reduced LVEF. First, it would be more comprehensive to include patients with preserved eject fraction to evaluate the effect of functional training. Second, the cardiopulmonary exercise test may offer more parameters to fully evaluate the fitness of the patient. Third, some elderly patients are not familiar with using mobile phones, another way may be clinically helpful.

## 6. Conclusions

Six months of post-acute HF focused on HCTR programs was safe, improved functional capacity, cardiac function, and decreased readmission rate in elderly patients with HF patients. Our study provided some evidence that home telemonitoring could represent a promising tool for older HF patients, ensuring that the benefits induced by physical activity remain longer in time. More studies are needed to give more precise information about the potential clinical effectiveness of home telehealth interventions.
